# Non-exercise activity thermogenesis (NEAT): a component of total daily energy expenditure

**DOI:** 10.20463/jenb.2018.0013

**Published:** 2018-06-30

**Authors:** Nana Chung, Mi-Young Park, Jisu Kim, Hun-Young Park, Hyejung Hwang, Chi-Ho Lee, Jin-Soo Han, Jaemoo So, Jonghoon Park, Kiwon Lim

**Affiliations:** 1 Physical Activity and Performance Institute (PAPI), Konkuk University, Seoul Republic of Korea; 2 Department of Physical Education, Konkuk University, Seoul Republic of Korea

**Keywords:** Obesity, energy expenditure, non-exercise activity thermogenesis (NEAT), sitting disease

## Abstract

**[Purpose]:**

The purpose of this review is to promote awareness of non-exercise activity thermogenesis (NEAT) as a new strategy to increase energy expenditure, and to manage obesity.

**[Methods]:**

The content of this review is based on a literature search of PubMed and the Google Scholar search engine, using the search terms obesity, energy expenditure, non-exercise activity thermogenesis (NEAT), and sitting disease.

**[Results]:**

Daily energy expenditure is of great interest because most obese individuals have no exercise activity-related thermogenesis (EAT); thus their physical activity-related energy expenditure (PEE) is comprised almost entirely of NEAT. Consequently, NEAT represents the main variable component of daily total energy expenditure (TEE); this varies considerably, both within among individuals. These somewhat unplanned and unstructured low level physical activities are associated with energy expenditure in excess of the resting metabolic rate (RMR). They may therefore have the potential to stimulate greater energy expenditure over time with a higher rate of adherence.

**[Conclusion]:**

In conclusion, NEAT is a highly variable component of daily TEE and a low level of NEAT is associated with obesity. NEAT enhances lifestyle, and variations in individual and environmental factors can significantly affect daily energy expenditure. Therefore, well designed longitudinal studies that focus on personal behavioral approaches and re-engineered environments to increase NEAT should be conducted in the future.

## INTRODUCTION

Obesity is a leading preventable cause of death worldwide; however obesity rates are increasing in adults and children^1^. Globally, 600 million adults and 100 million children were obese in 2015^2^. In 2013, the American Medical Association classified obesity as a disease^3^. Individuals are considered to be obese when their body mass index (BMI), a measurement obtained by dividing body weight by the square of height (kg/m²), exceeds 30 kg/m², with a BMI of 25–30 kg/m² defined as overweight^1^. Some East Asian countries use lower values^4^. Excessive body weight increases the likelihood of various diseases, particularly type 2 diabetes mellitus, hypertension, dyslipidemia, cardiovascular diseases, obstructive sleep apnea, asthma, osteoarthritis and certain types of cancer^5-7^. As a consequence, obesity has been found to reduce life expectancy. Obesity poses serious public health and policy problems because of its prevalence, health effects and health-care costs^8^.

The etiology of obesity is multifactorial; resulting from genetic^9-11^, epigenetic^11,12^, physiological^13,14^, behavioral, sociocultural^15^, and environmental^16,17^ factors that lead to an imbalance between energy intake and energy expenditure. A limited case are genes, endocrine disorders, medications, or psychiatric disorder^18^. Excessive food intake and a lack of physical activity are believed to be the two most important causes of the recent increase in obesity^19^. The modern lifestyle with decreasing levels of physical activity and food intake in excess of daily energy expenditure is closely related to the increase in obesity. Nevertheless, whether increased energy intake is associated with the increased prevalence of obesity remains controversial, since previous studies have shown that energy intake has remained relatively constant. For example, in Britain since the 1980’s, obesity rates have doubled but energy intake appears to have actually declined^20^. Weinsier et al. 1998^21^ also found diverging trends of decreasing energy intake and increasing body weight. Furthermore, Hill et al. 2012^22^ speculated that if physical activity decreased, body weight may increase if energy intake is not altered. In the absence of evidence that positively associates increased energy intake with obesity, the role of physical activity in human energy balance has come under greater scrutiny. Since a reduction in energy expenditure might be the most important factor explaining the rising obesity pandemic^23,24^, it is important to examine the progressive decline in daily energy expenditure.

Traditional interventions to overcome a net positive energy balance have focused on encouraging moderate- to vigorous- intensity physical activity. However, this strategy has met with limited success in long-term randomized clinical trials. Therefore, a new strategy has recently emerged that focuses on increasing low level daily physical activity or NEAT (non-exercise activity thermogenesis), which includes the addition of short repetitive bouts of non-exercise physical activity. NEAT demonstrates inter-individual variation due to differences in occupation and leisure-time activities; however it is crucial to the regulation of energy expenditure. Therefore NEAT may contribute to our understanding of the causes and successful treatment of obesity.

The purpose of this review is to promote NEAT as a new strategy to increase energy expenditure and to aid the management of obesity.

## METHODS

This review used literature obtained from the PubMed and Google Scholar search engines, with the following search terms: obesity, energy expenditure, non-exercise activity thermogenesis (NEAT) and sitting disease. We conducted a comprehensive literature search from inception up to 2018, without language or year of publication restrictions. We excluded studies with incomplete data, letters, editorials and case reports. The references of the studies included in the full-text review were searched for cross-references, to find the studies that could have been missed in the original search.

## RESULTS and DISCUSSION

### NEAT: a component of total daily energy expenditure

Three main components of energy balance determine total energy expenditure (TEE): resting metabolic rate (RMR), diet-induced thermogenesis (DIT), and physical activity-related energy expenditure (PEE)^25^.

RMR represents the minimal amount of energy expended for homeostatic processes^23^,^24^, and it accounts for approximately 60% of daily total energy expenditure (TEE) in a mainly sedentary individual^26^. About 80% of the variance in RMR is determined by body size, with lean body mass (LBM) positively correlated with RMR. Diet-induced thermogenesis (DIT) accounts for approximately 10-15% of TEE, and it is related to digestion, absorption and the storage of food. The variance of DIT has been associated with the nutrient composition and energy content of consumed foods^27^. Although it is theorized that energy expenditure changes in relation to alterations in intake, the magnitude of change remains disputed^28,29^, and it does not vary greatly between individuals^30^. The third main determinant of TEE is physical activity-related energy expenditure (PEE) that accounts for 15% to 30% of TEE. It is defined as the additional energy expenditure above RMR and DIT, which is required for activity. PEE can be further subdivided into exercise-related activity thermogenesis (EAT) and NEAT. These vary widely, both within and among individuals. EAT is defined as planned, structured, and repetitive physical activity that has the objective of improving health (for example, sport, visiting the gym)^31^. NEAT represents the predominant component of daily activity thermogenesis with the exception of some sports-like exercise and resistance training^32^. NEAT is the energy expenditure that we do not typically consider and it includes the energy expended maintaining and changing posture (laying, standing, walking, stair climbing, spontaneous muscle contraction, fidgeting, cleaning), singing, and other activities of daily living. These activities do not involve moderate- to vigorous- intensity exercise and occur at a trivial or a low energy workload for minutes to hours.^33,34^. These somewhat unplanned and unstructured low grade physical activities can have a remarkable effect on metabolic rate and, as a result, stimulate greater energy expenditure over time.

### Causes of decreased energy expenditure

Basal metabolic rate (BMR) is largely (~80%) accounted for by body size and DIT is a relatively small component of TEE (10-15%), so the variance in TEE between individuals of similar size can only be explained by variance in PEE (EAT and/or NEAT)^26,29,32^. Recently, there has been a large shift towards less physically demanding work, and currently at least 30% of the world's population lacks sufficient physical activity^1^. This is primarily due to an increasing reliance on cars, mechanized manufacturing and a greater prevalence of labor-saving technology in the home, combined with an inactive lifestyle^35^. This has caused a progressive and systematic decline in energy expenditure, resulting in a sustained positive energy balance. More time is devoted to sedentary behaviors involving prolonged sitting, and this global trend is likely to continue. Obesity has emerged as a result of environmental influences with increasing inactivity. Evidence suggests that an active lifestyle conveys many important health benefits, and sedentary habits are associated with an increased risk of chronic diseases and decreased life expectancy^36^. Recognition of the health and functional hazards of an inactive lifestyle has led to the development of public health recommendations for physical activity.

### Traditional strategies to increase energy expenditure

Low energy expenditure was suggested to play a role in the development of obesity because of a lower RMR, DIT, PEE or a combination of all of these components, contributing towards a positive energy balance and subsequent weight gain^37^. For obesity treatment to be successful, an understanding of the physiological determinants of obesity should be carefully considered. Although our understanding of body weight regulation and energy balance has evolved, how alterations of the individual components of energy expenditure influence energy balance is debated. Nevertheless, PEE (EAT + NEAT) has the greatest variability of all the TEE components.

To address obesity and its related clinical complications, strategies that are mainly based on physical activity in the form of EAT have been implemented. To prevent and control obesity, exercise guidelines typically recommend > 30 min/day of moderate intensity exercise, at least 5 days/week^38-40^. There is sufficient exercise physiology research to support these public health guidelines, promoting at least 150 min/week of moderate-vigorous leisure-time physical activity, aimed at decreasing the risk of metabolic diseases^41,42^. Commonly EAT accounts for a maximum of 15-30% of TEE in those who regularly participate in the recommended physical training^25,43^, and it explains 1-2% of the variance in TEE. However, for the majority of people in modern society, EAT is believed to be negligible. Also, adherence to the recommended exercise intensity and duration remains low in obese patients, and consequently, EAT is nearer to zero^26,44^. Even for people who participate in exercise for less than two hours a week, exercise accounts for an average energy expenditure of only~100 kcal/day^45^. Interestingly, even if adults adhere to the exercise guidelines, sitting for prolonged periods of time can compromise their metabolic health^46^. Therefore, NEAT has emerged as the main component of TEE variability^30^. This suggests that in addition to moderate- vigorous intensity physical activity, light-moderate intensity daily physical activity should also be considered as an alternative and complimentary exercise therapy regimen for obese individuals. Attention should shift to an active lifestyle intervention to reduce obesity, with NEAT considered as a substitute for the amount of time currently spent inactive, to increase the low level of energy expenditure.

### NEAT as a new strategy to increase energy expenditure

If the EAT of individuals with obesity is considered to be negligible, PEE is comprised entirely of NEAT. Consequently, NEAT represents the main variable component of TEE within individuals, and it also varies substantially between individuals. Although the contribution of NEAT to daily energy expenditure is negligible, it can accumulate over time, eventually becoming an important factor in weight loss or weight gain. The variability in work, leisure-time and household activities between individuals plays a fundamental role in NEAT differences^46-50^. Thus, the biggest advantage of NEAT is that the requirement to comply with purposeful exercise is low, and it has a higher rate of adherence over time. This was observed in a Chinese study that compared the prevalence of recommended exercise and NEAT in 32 005 adolescents; NEAT remained high over time and seemed easier to accumulate than exercise in adolescents, regardless of age or gender^51^.

Daily additive unplanned or unstructured low intensity activities are associated with energy expenditure in excess of the RMR and account for significant thermogenesis and energy expenditure^52,53^. The metabolic equivalent (MET) is a useful measurement to represent the intensity of physical activity and it is defined as the amount of oxygen uptake while sitting at rest. An oxygen uptake of 3.5 mL/kg/min is equal to the RMR and is considered to be equivalent to 1.0 MET^54^. Sitting, standing and walking increase energy expenditure above resting levels by 5-10%, 10-20% and 100-200%, respectively^55-58^. Ainsworth et al. 2011^59^ reported that walking inside is equal to only 2.0 METs, whereas walking with children is equivalent to 4.0 METs. Likewise, daily physical activity can span a wide range of intensity levels that at times are the same as structured exercise.

In fact, energy expenditure induced by NEAT is much larger than EAT when measured over the day^60,61^. Levine et al., 1999^60^ observed that long-term weight control may be easier to maintain by focusing less on EAT and more on increasing NEAT. They recruited 10 lean and 10 mildly obese sedentary volunteers and measured their postures, activities of daily living, and fidgeting for 10 days. Obese individuals were seated on average, two hours a day longer than lean people. If obese individuals adopted the NEAT-enhanced behaviors of their lean counterparts, they could expend an additional 350 kcal/day from these numerous small low grade activities and movements. Since this is equivalent to approximately 18 kg in a year, this may be an important factor in long-term weight control. Other studies have also demonstrated that obese individuals tend to have a lower level of NEAT than non-obese individuals, with more time spent engaging in sedentary activities (for example, laying down, sitting, watching television)^62-64^. Before increasing NEAT is proposed, the number of calories which need to be expended to prevent or treat obesity should be considered. According to Levine et al. 2007^61^, an additional energy expenditure of 280-350 kcal/day or 2000-2500 kcal/week through NEAT is required for weight loss. However, there is lack of evidence from randomized controlled studies to support whether strategies promoting NEAT are effective for obesity treatment, and there is limited research examining the thermogenic potential of fidgeting-like activities at very low workloads. In addition individual biological and environmental factors such as gender, age, body composition, and season can result in significant variation in NEAT. These four factors will therefore be discussed in more detail. 1) Gender is biologically determined but there are gender-specific environmental cues that influence NEAT. Although the United States report similar levels of PEE in adult men and women^65^, in other countries, including Canada, England, and Australia, men tend to have a higher PEE than women^66^. Gender may also influence NEAT in more subtle ways. For example, society and culture may require that women to do more housework and child care. 2) Previous studies have demonstrated an age-related decline in NEAT^67^. Harris et al. 2007^68^ showed a substantial decrease in NEAT in elderly compared to younger individuals. Indeed, elderly people showed 29% less NEAT, and this result was mainly attributable to the NEAT subcomponent of ambulation. 3) Overweight individuals tend to have lower NEAT levels than their lean counterparts^69,70^, and this is consistent across all ages, for both genders. It is not possible to ascertain whether the effects of body composition on NEAT are independent of weight ^32^. 4) Limited data are available regarding differences in NEAT across the seasons; however, physical activity time is twice as high during the summer compared with the winter^26^. These data were explained by the seasonal variation in occupation- associated NEAT^71-73^. For example, there is greater emphasis on agricultural work or work in the construction industry during the summer. In addition, people do more outdoor activities in the summer. Therefore, further research is required to accurately measure both locomotor and household activities, and to further our understanding of NEAT in the different stages of life, from childhood to early and late adulthood. Since movement patterns during these unique periods differ, NEAT recommendations may be specific for each age group. However, at present, the general advice should be to encourage people to limit their sitting time whilst at home, at work, and using transport. Prolonged periods of sitting throughout the day should be interrupted by frequent transitions from sitting to standing and ambulation^74^.

### The metabolic health consequences of NEAT

Recently studies have shown that metabolic syndrome^75-77^, adiposity or weight gain^45,78-80^, poor glucose management^81^ and type 2 diabetes risk^82,83^ may be directly related to sitting time and/or to a low level of NEAT that may be independent of EAT. Such findings suggest that sedentary behavior that does not increase energy expenditure substantially above resting levels, may contribute to disease risk separately from EAT, affecting health via independent pathways. Therefore, EAT does not counteract the negative effects of prolonged sitting. This is supported by a Canadian study which showed a dose-response relationship between sitting time and all-cause mortality and cardiovascular disease (CVD) mortality, independent of EAT. After adjusting for cofounders, they found a progressively higher risk of all-cause mortality and CVD mortality with higher levels of sitting time. Moreover, Hagger-Johnson et al.^84^ analyzed data from the United Kingdom Women’s Cohort Study (1999-2002) to investigate the association between fidgeting behaviors and allcause mortality. They found that subjects in the low fidgeting group, sitting for ≥ 7 h/day (vs. < 5 h/day) experienced a 30% increase in risk of all-cause mortality. In contrast, in a large scale cohort study, Wen et al., 2011^85^, found that individuals who performed low volume physical activity, defined as 15 min/day or 90 min/week, had a 14% reduced risk of all-cause mortality and a three year increase in life expectancy. Thus, in addition to EAT through moderate- to vigorous- intensity exercise, light- to moderate- intensity daily NEAT should also be considered as an alternative and complimentary therapy regimen to improve metabolic health. Nevertheless, research in NEAT-related fields is in the early stage, and uncertainty still exists regarding which components of energy expenditure are altered in obese patients and patients with metabolic diseases. The documented effects of the different components of energy expenditure on TEE in obese patients and patients with metabolic diseases are inconsistent. Thus, it is necessary to accumulate evidence on the positive effects of EAT on the management of obesity and metabolic diseases.

### NEAT quantification

At present, several methods are used to measure energy expenditure. Indirect and direct calorimetric and non-calorimetric methods are used for providing information on body posture and activity recognition. Questionnaires can provide qualitative information, such as the type and the purpose of physical activity, which differs to that provided by objective methods. However, questionnaires are unable to accurately measure the intensity of physical activity and energy expenditure. The NEAT score calculated using a questionnaire is subjective and may not always represent the true NEAT^86-88^. In contrast, instruments such as accelerometers will provide more accurate information on the intensity, duration, and frequency of PEE, mitigating the recall bias of self-report questionnaire data^89^. Also, accelerometers in mobile phones and stand-alone devices with consumer applications enable precise and accurate measurement of daily physical activity^90^. These are attractive strategies for encouraging the measurement of physical activity and they are easy to self-monitor and user-friendly^91^. Nonetheless, this method has the disadvantage that physical activity can only be measured when the mobile phone is on the body, and it does not accurately reflect actual energy expenditure in situations such as walking carrying objects, and walking uphill^88,91^. Methods of measuring energy expenditure based on the concentration of oxygen and carbon dioxide are relatively accurate, however conventional indirect calorimetry equipment configurations either prohibit movement completely (for example, hood calorimetry), or involve the application of a mouthpiece and nose clip that may prevent normal body movements, (in this case breathing may differ to normal). The doubly labeled water (DLW) method is one of the best techniques for measuring TEE under free-living conditions, however, this method can only evaluate TEE and it cannot assess variations in the TEE components^83^. Thus, separate measurements of NEAT are limited by the need to measure activity level using an accelerometer. The human metabolic chamber (HMC), is an indirect calorimetric method that can overcome these limitations and it can be used to measure all of the components of TEE, including RMR, DIT, EAT, and NEAT. This equipment also allows the precise measurement of energy expenditure with unrestricted movement and a rapid response time. Therefore, it is considered to be a valuable method in this research field.

## CONCLUSION

In conclusion, NEAT is a highly variable component of daily TEE and a low level of NEAT is associated with obesity. NEAT enhances lifestyle, and variations in individual and environmental factors can significantly affect daily energy expenditure. Therefore, well designed longitudinal studies that focus on personal behavioral approaches and re-engineered environments to increase NEAT should be conducted in the future. A greater understanding of the amounts and intensities of NEAT needed to protect against obesity and metabolic diseases in the context of gender and age differences is required. Also, the feasibility of NEAT regarding the thermogenic potential of fidgeting-like activities at very low workloads for different groups (older, younger), in different settings (workplace, domestic, transit), needs to be explored. Finally, intervention trials are required to demonstrate whether significant increases in NEAT are associated with improvements in the relevant biomarkers.
